# Ultra-High-Performance Liquid Chromatography–Orbitrap-MS-Based Untargeted Lipidomics Reveal Lipid Characteristics of a Clinical Strain of *Mycoplasma bovis* from Holstein Cow

**DOI:** 10.3390/vetsci11110577

**Published:** 2024-11-18

**Authors:** Fei Yang, Mengmeng Yang, Fan Liu, Yanrong Qi, Yanan Guo, Shenghu He

**Affiliations:** 1 Institute of Animal Sciences, Ningxia Academy of Agricultural and Forestry Sciences, Yinchuan 750002, China; 12021140116@stu.nxu.edu.cn (F.Y.); 20150155@nxmu.edu.cn (M.Y.); 2 College of Animal Science and Technology, Ningxia University, Yinchuan 750021, China; 12022140132@stu.nxu.edu.cn (F.L.); 12022131403@stu.nxu.edu.cn (Y.Q.); 3 Agricultural and Rural Bureau of Helan County, Ningxia Hui Autonomous Region, Yinchuan 750200, China

**Keywords:** *Mycoplasma bovis*, untargeted lipidomics, glycerophospholipid metabolism

## Abstract

*Mycoplasma bovis* is a global concern for the cattle industry owing to its high rates of infection and resulting morbidity, but there are no reports on the lipid composition and metabolic pathways. This study examined the lipidomics of *M. bovis* to better investigate the biological characteristics of clinical isolates of *M. bovis*. The *M. bovis* strains NX114 and PG45, cultivated to log-phase, underwent untargeted lipidomics via ultra-high-performance liquid chromatography-mass spectrometry for differential analysis. Over 65 lipid types and 1476 lipid molecules were identified. We found that glycerophospholipids and sphingolipids constitute the predominant lipid components of *M. bovis*, with significant constituents of its cell membrane comprising ceramides, phosphatidylglycerols, sphingomyelins, triacylglycerols, hexosylceramides, ether-linked oxidized phosphatidylcholines, and oxidized phosphatidylglycerols. Secondly, 562 differentially expressed lipid molecules were identified, including 17 lipid subclasses (15 up-regulated and 2 down-regulated) with significant differences in relative content. These findings indicate that distinct *M. bovis* isolates contain structurally varied lipid molecules, with sphingomyelin, phosphatidylinositol, cardiolipin, and phosphatidylcholine being characteristic lipids. The glycerophospholipid metabolism pathway was identified as a key pathway regulating lipid metabolism in *M. bovis* by KEGG pathway enrichment analysis. The results indicated alterations in the lipid metabolism of *M. bovis*, offering insights into its pathogenic mechanisms.

## Simple Summary

*Mycoplasma bovis* is a pathogenic microorganism that is easily overlooked but extremely harmful to cattle. In this study, we analyzed non-target lipids of *M. bovis* clinical strain NX114 and international standard strain PG45 as a control. We discovered important components of *M. bovis* lipids and further expanded its bioinformatics. The presence of structurally diverse differential lipid molecules in different isolates of *M. bovis*. Explore the important lipid metabolic pathways during the metabolism of *M. bovis*. These are important for further unraveling the lipid-associated pathogenesis of *M. bovis* and for developing new anti-pneumonia drugs and attenuated vaccines.

## 1. Introduction

*Mycoplasma bovis* is a pathogenic microorganism, easily overlooked, but extremely harmful to cattle. It can lead to a variety of bovine diseases, such as pneumonia, mastitis, arthritis, otitis media, and corneal conjunctivitis, and thus may result in great economic losses to the global cattle industry [1,2]. Thus far, there is no effective vaccine to prevent *M. bovis* infection. In addition, the evolution of antibiotic resistance poses a significant obstacle to the treatment of this disease, making both its prevention and control exceedingly challenging [3,4].

Lipids are a class of large hydrophobic or amphoteric small molecules that form and maintain the membrane structure of organisms, as well as function as signal molecules and energy substances [5]. Lipidomics is a systemic analytical biology research method that uses high-throughput analysis and quantitative techniques to study changes in lipid composition and expression patterns, enabling the efficient study of lipid family and molecule functions in various biological processes. Lipidomics has resulted in significant advancements in the elucidation of the physiological and pathological roles of lipids through its focus on metabolic phenotype analysis [4,6]. Currently, liquid chromatography–mass spectrometry (LC–MS) is commonly used for lipidomics analysis, categorized into the untargeted and targeted analysis.

Untargeted lipidomics is a comprehensive and unbiased analysis of all lipids in extracted samples, conducted in the absence of information about the target lipid molecules [5,7,8]. The method first examines the characteristics of “all” ions in the sample, including precise mass (*m*/*z*), retention time, and peak area; screens for differences in ions by multivariate statistics; and then searches for precise mass information or secondary spectra for identification of lipid molecules (reference molecules) by using databases such as the Human Metabolome Database (HMDB), Lipid Blast, and Lipid Maps. The information is searched for the precise mass information or secondary spectra of the ions for identification and to obtain lipid molecules [9]. Non-targeted lipidomics focuses on the comprehensive analysis of lipids, and in addition to the discovery of unknown lipid molecules, its large amount of detailed lipid profiles can be used clinically for assessing medical risks and monitoring and optimizing patient treatments, which is the basis of the concept of precision medicine [10]. In addition, targeted lipidomics is the accurate identification and quantification of target lipids for which standards are available, with greater accuracy and sensitivity [11]. Multiple reaction monitoring (MRM) or parallel reaction monitoring (PRM) modes are the main quantitative modes in targeted lipidomics [12], and ultra-high-performance liquid-phase tandem triple quadrupole mass spectrometry (QMS) is a commonly used instrument for targeted lipidomics [13], which has the advantage of analyzing most of the lipids in biological samples with a wide linear range, high sensitivity, and stability [14]. Targeted lipidomics is a purposeful approach to studying certain lipid molecules, usually after setting a specific research goal and selecting associated lipid molecules for analysis, which helps to gain insight into the role of lipid molecules in physiological and pathological processes [15].

*M. bovis* is a small, simple, self-replicating prokaryotic microorganism that exists in nature between bacteria and viruses. Our previous non-targeted metabolomics analyses revealed differences in growth phenotype and metabolic profiles between *M. bovis* NX114 and PG45 [16]. We also identified the presence of key genes regulating the growth of *M. bovis* using combined transcriptome–proteome analyses [17]. However, the lipid profiles of *M. bovis* have not been fully analyzed. This study utilized ultra-high-performance liquid chromatography (UHPLC)–Obitrap MS technology for lipid profiling of non-target lipids from the clinical isolate NX114 and an international standard strain PG45 from various dairy cow parts. We identified the major components of lipids in *M. bovis*. Significant differential lipids were screened out, followed by cluster analysis, correlation analysis, trend change analysis of lipid categories, and KEGG pathway enrichment analysis of differential lipid molecules. This high-throughput lipidomic approach can be used to better understand the lipid changes associated with the pathogenicity of *M. bovis*.

## 2. Materials and Methods

### 2.1. Bacterial Strains and Media

The clinical strain NX114 (GenBank accession no. CP135997) of *M. bovis* was obtained from a Holstein cow with pneumonia in Ningxia Province, China. The international standard strain PG45 was provided by Professor Aizhen Guo from Huazhong Agricultural University. Both strains were preserved in PPLO broth containing 40% glycerol at −80 °C in a laboratory and cultivated in PPLO broth before use [18].

### 2.2. Transmission Electron Microscopy

Samples were serially diluted at about 1:10 with DPBS (without Ca^2+^/Mg^2+^) and visualized by 1% phosphotungstic acid negative staining as follows. A 15µL aliquot was added to a grid with a formvar supporting film coated with carbon for 5 min. The excess solution was soaked off with filter paper, and the samples were stained for 3 min and air-dried. Samples were visualized using an electron microscope (JEM-1400 FLASH, JEOL, Tokyo, Japan) at 80 kV. Digital images were taken with a camera (Germany, EMSIS, Münster, Germany).

### 2.3. Extraction of Lipids from M. bovis Strains

Liquid samples (400 μL) of *M. bovis* were taken during the logarithmic phase [16], combined with 75% methanol (100 μL), and sonicated on ice for 15 min. Then, 1 mL of methyl tert-butyl ether was added, and the sample was vortexed on ice shock. The sample was placed in the refrigerator at 4 °C and blended for 1 hr using a rotator, followed by sonication on ice for 15 min (2 times). Then, the sample was combined with 200 μL of water, vortexed for 1 min, left at room temperature for 10 min, and centrifuged at 14,000× *g* for 15 min at 4 °C. The supernatant was removed, and the precipitate was placed in a fume hood to air-dry, then 200 μL of SDT was added, and the protein concentration was quantified using the bicinchoninic acid assay (BCA). The concentration in each sample was adjusted based on the protein concentration, and a volume of the lipid fraction containing an equal amount was taken from each example sample and dried by volatilization using a nitrogen blower. The solution was redissolved in 40 μL isopropanol/methanol (1/1, *v*/*v*), transferred to an injection bottle, centrifuged at 20,000× *g* at 4 °C for 20 min, and the supernatant was injected for analysis.

### 2.4. UHPLC–MS/MS Analysis

Samples were analyzed on a SHIMADZU-LC30 ultra-high-performance liquid chromatography system using an ACQUITY UPLC^®^ HSS C18 column (2.1 × 150 mm, 2.5 µm; Waters, Milford, MA, USA). The injection volume was 5 μL, the column temperature was 40 °C, and the flow rate was 0.3 mL/min. The mobile phase consisted of A, ammonium formate in acetonitrile/water (6:4, *v*/*v*), and B, mobile phase of acetonitrile and isopropyl alcohol (1:9, *v*/*v*). The solvent gradient changed according to the following conditions: linear gradient from 30% to 32% B, 0–2 min; from 32% to 45% B, 2–6 min; from 45% to 52% B, 6–8 min; from 52% to 58% B, 8–12 min; from 58% to 66% B, 12–15 min; from 66% to 70% B, 15–18 min; from 70% to 97% B, 18–21 min; hold at 97% B, 21–25 min; from 97% to 32% B, 25–26 min; hold at 32% B, 26–30 min. During the whole analysis process, the samples were placed in the autosampler at 4 °C. In order to avoid the influence caused by the fluctuation of the instrument detection signal, the samples were continuously analyzed in random order. Each group of samples was equipped with 6 replicates for a total of 12 samples, and 1 quality control (QC) sample is set for every 4 samples in the sample cohort, for a total of 3 QC samples, which are used for monitoring and evaluating the stability of the system and the reliability of the experimental data.

Electrospray ionization (ESI) was used to detect positive ions and negative ions after UHPLC separation using Q Exactive Plus mass spectrometer (Thermo Scientific™) mass spectrometry analysis. The parameter settings were probe heater temperature 300 °C; sheath gas flow rate, 45 arb; auxiliary gas flow rate, 15 arb; sweep gas flow rate, 1 arb; spray voltage, 3.0 kv (ESI+) or 3.5 kv (ESI−); capillary temperature, 350 °C; S-lens RF level, 50%; and scan ranges, 200–1500.

### 2.5. Multivariate Data Processing and Analysis

The software MS-DIAL (Version 4.9.0) was used for lipid identification and quantification. The main parameters were 10 ppm precursor mass tolerance and 10 ppm product mass tolerance; a retention time of 0.1 min for ion peak alignment; chromatographic mass spectrometry peak area was used to reduce the false positive extracted data; and the ion peak area was removed from the QC samples. The peak area of the chromatogram was used to reduce the false-positive extracted data, and the lipid molecules with an RSD > 30% in the QC sample were removed.

The detailed steps include: (1) peak extraction: using MS-DAIL software that involved reading ABF files from raw data exported by Q Exactive LC–MS/MS, and reading the MSn and the exact mass number of the parent ion; (2) identification: based on the parent ion and multistage mass spectrometry data in each independent sample and identify the structure of the lipid molecules therein and their positive and negative ion addition patterns; (3) peak alignment for each independent sample by aligning the search results according to a certain retention time range and combining results into a single report that organizes the raw data matrix. This three-dimensional matrix included information such as sample information, identification of each substance peak, lipid class, branched-chain information, retention time, mass-to-charge ratio, and mass spectral response intensity (peak area).

The data extracted by MS-DIAL software were normalized to the total peak area, and the data were preprocessed by unit variance (UV) scaling for multidimensional statistical analyses, including unsupervised principal component analysis (PCA) analysis, supervised platoon least squares discriminant analysis (PLS-DA), and orthogonal partial least squares discriminant analysis (OPLS-DA). The evaluation parameters of the OPLS-DA model (R2Y, Q2) were obtained by 7-fold cross-validation, and with R2, Q2 ≥ 0.5, the model was stable and reliable. In order to avoid overfitting of the supervised model in the modeling process, a permutation test was used to test the model to ensure its validity; when the intercept of the Q2 regression straight line with the y-axis (Q2intercept) was < 0.05, the model did not have an overfitting phenomenon. Using the OPLS-DA obtained by the variable projected importance (VIP) > 1 model and the difference multiplier FC > 1.5 or FC < 0.667 as the screening criteria, significantly differential lipids were obtained. Software (version 1.6.2) was used to draw volcano diagrams and to perform various analyses, including hierarchical clustering and correlation analysis. We used the LIPEA tool for pathway enrichment analysis of differential lipids and KEGG ID mapping of differential lipid molecules in the comparison group.

## 3. Results

### 3.1. Morphological Observations of M. bovis

**Figure 1:**
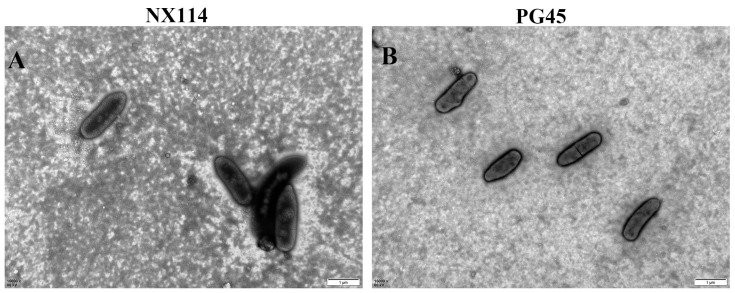
Examination of the morphology of *M. bovis* by transmission electron microscopy after negative staining. (**A**) NX114 (15,000×); (**B**) PG45 (15,000×).

Transmission electron microscopy showed that the shape of *M. bovis* was oval and irregularly polygonal, and its size was varied and not consistent. The cells of the clinical isolate of *M. bovis* NX114 (Figure 1A) were slightly larger than those of the standard strain PG45 (Figure 1B).

### 3.2. Lipid Content and Composition of M. bovis

**Figure 2:**
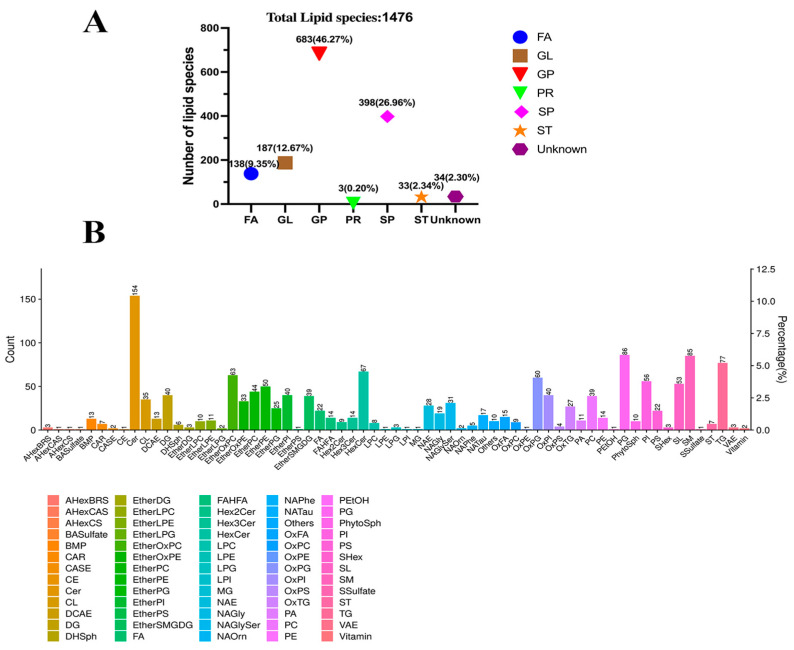
Lipid composition in *M. bovis*. (**A**) Statistical distribution map of the number of lipid major classes. FA: fatty acid, GL: glycerolipids, GP: glycerophospholipid, PR: prenol lipid, SP: sphingolipid, and ST: sterol lipid. (**B**) Graph of statistical distribution of lipid subclasses and number of lipid molecules. The *x*-axis is subclasses of lipids; the left *y*-axis is the number of lipids; and the right *y*-axis is the percentage. Cer: ceramide, PG: phosphatidylglycerol, SM: sphingomyelin, TG: triacylglycerol, HexCer: hexosylceramide, EtherOxPC: ether-linked oxidized phosphatidylcholines, OxPG: oxidized phosphatidylglycerols, PI: phosphatidylinositol, SL: sphingolipids, EtherPE: ether phosphatidylethanolamine, EtherPC: ether phosphatidylcholine, EtherPI: ether phosphatidylinositol, OxPI: oxidized phosphatidylinositol, DG: diacylglycerol, and PC: phosphatidylcholine (top 15).

**Figure 3:**
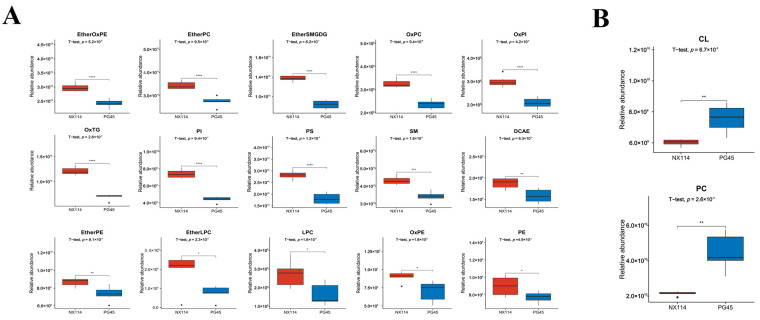
Comparative analysis of the relative content of lipid group subclasses in the *M. bovis* NX114 vs. PG45 strains. (**A**) Relative abundance of significantly up-regulated lipid group subclasses. (**B**) Relative abundance of significantly down-regulated lipid group subclasses. (Significance is shown using Welch’s *t*-test, with the specific *p*-value noted in the graph and also denoted by asterisks (*, *p* < 0.05, **, *p* < 0.01, ***, *p* < 0.001, ****, *p* < 0.0001); ns, no significance (*p* > 0.05).

Lipids in *M. bovis* identified based on UPLC-Q-Exactive Orbitrap MS consisted of 6 major classes of lipids and 34 unknown lipid molecules comprising classes, of which glycerophospholipids (GP) and sphingolipids (SP) were the major components, accounting for 46.27% (683/1476) and 26.96% (398/1476) of the lipid species, respectively (Table S1; Figure 2A). The 1476 lipid species of *M. bovis* fall within 65 lipid subclasses and include 154 ceramides (Cers), 86 phosphatidylglycerols (PGs), 85 sphingomyelins (SMs), 77 triglycerides (TGs), 67 hexosylceramides (HexCers), 63 species of ether-linked oxidized phosphatidylcholines (EtherOxPCs), and 60 species of oxidized phosphatidylglycerols (OxPGs) (Table S2; Figure 2B). Among them, ether-linked PCs (EtherPCs), DHSph, Cer, and PG species accounted for a relatively high proportion of the total kurtosis of lipid subclasses in the two strains of *M. bovis* (Figure S1). In addition, comparative analysis of the relative content of lipid group subclasses in the NX114 vs. PG45 group revealed significant differences in the relative content of 17 out of 65 lipid subclasses (15 up-regulated and 2 down-regulated), comprising up-regulation of EtherOxPE, EtherPC, OxPC, OxPI, PI, PS, EtherSMGDG, and OxTG (*p* < 0.0001), SM (*p* < 0.001), EtherPE, and DCAE (*p* < 0.01), EtherLPC, LPC, OxPE, and PE (*p* < 0.05) (Figure 3A), and downregulation of cardiolipin (CL) and PC (*p* < 0.01; Figure 3B).

### 3.3. Multidimensional Statistical Analysis of Lipid Molecules of M. bovis

**Figure 4:**
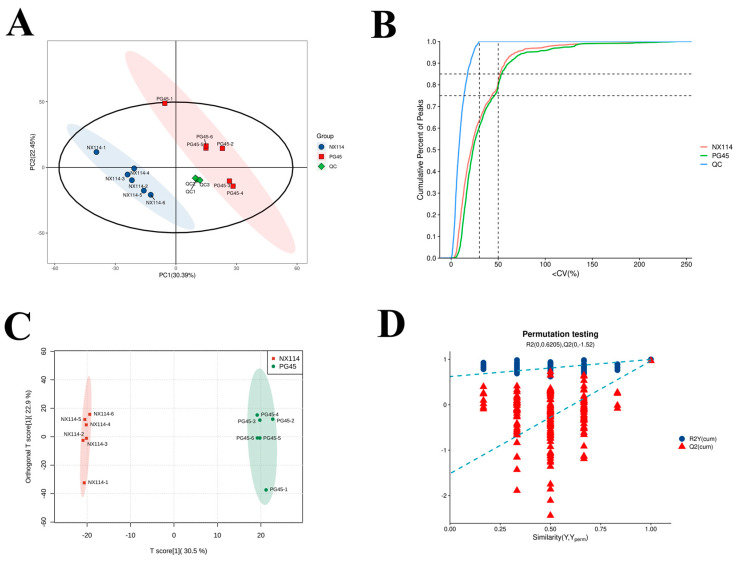
Multidimensional statistical analysis of lipid profiles comparing the *M. bovis* NX114 vs. PG45 strains. (**A**) Plot of PCA scores. (**B**) Cumulative curve of the coefficient of variation for the data. (**C**) Plot of OPLS-DA scores. (**D**) Plot of OPLS-DA permutation tests. R2Y: denotes the model explanation rate; Q2: denotes the model prediction ability; the closer R2Y and Q2 are to 1, the more stable and reliable the model; R2 intercept and Q2 intercept: denote the intercept of R2 and Q2 regression lines with the y−axis; and when the Q2 intercept is <0.05, the model does not have the phenomenon of overfitting (R2X(cum) = 0.534, R2Y(cum) = 0.998, Q2(cum) = 0.967, R2 Intercept = 0.621, Q2 Intercept = −1.517).

In this study, the peaks obtained from the extraction of all samples and QC samples were analyzed by PCA after UV scaling, and the two groups of samples showed a clear trend of separation on the PC1 and PC2 dimensional plots. The PCA model was obtained after 7-fold cross-validation (Figure 4A). The coefficient of variation (CV value) was calculated for each metabolite in the QC samples and for the overall data; a high cumulative percentage of metabolites with a CV *<* 0.3 indicates less fluctuation during the experiment (Figure 4B). We established the OPLS-DA model for the comparison of NX114 vs. PG45, which showed that the OPLS-DA model could distinguish between the two groups of samples (Figure 4C). The model evaluation parameters (R2Y, Q2) obtained by 7-fold cross-validation, established by the data of the comparison group in this experiment, R2, Q2 ≥ 0.5, showed that the OPLS-DA model was stable and reliable. In order to avoid overfitting of the supervised model in the modeling process, a replacement test (permutation test) was used to test the model, and the model was not overfitted when the intercept of the Q2 regression straight line with the y-axis (Q2intercept) was <0.05 in this experiment (Figure 4D).

### 3.4. Analysis of Differentially Expressed Lipid Molecules in M. bovis

**Figure 5:**
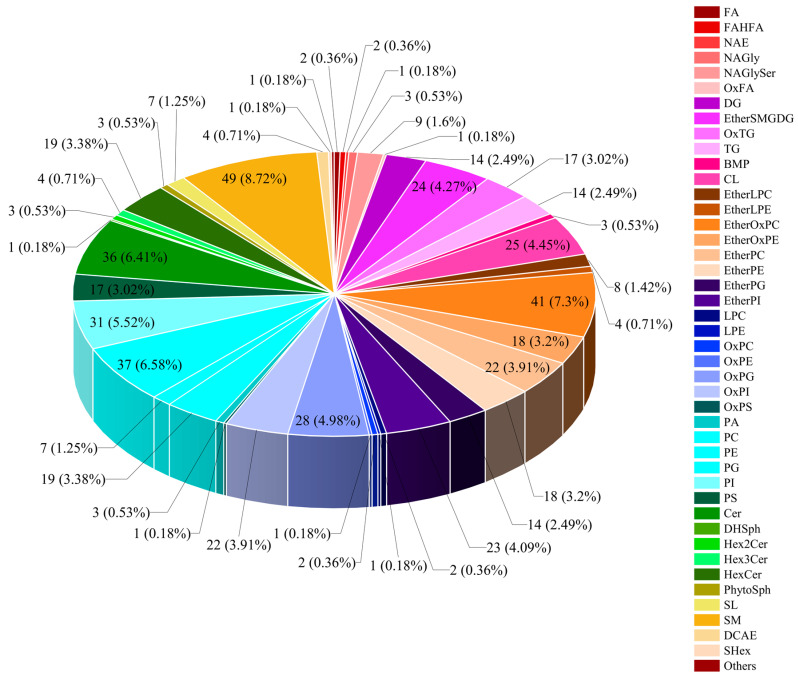
Distribution of significantly differentially expressed lipids in NX114 vs. PG45 strains of *M. bovis* (adjusted *p* < 0.05). Differences in the number of lipid macrophages. Differences in the number of lipid subclasses.

In this experiment, VIP > 1, *p* value *<* 0.05, and multiplicity of difference FC > 1.5 or FC *<* 0.667 in the model established by OPLS-DA were used as the criteria to screen for significant differentiators in the comparison group. The volcano plot demonstrates the different metabolites screened by univariate statistical analysis for the comparison group NX114 vs. PG45 (Figure S2). A total of 44 differential lipid subclasses and 562 differential lipid molecules, including 378 up-regulated and 184 down-regulated lipid molecules, were screened in the four major lipid classes (Table S3). Among them, GP and SP remained the major components of differential lipids in the M. bovis NX114 vs. PG45 comparison group, accounting for 61.74% and 21.71% of the differentially expressed lipids, respectively. The main ones included 49 species of SMs, 41 species of EtherOxPCs, 37 species of PGs, 36 species of Cers, 31 species of phosphatidylinositols (PIs), and other differential lipids (Figure 5).

**Figure 6:**
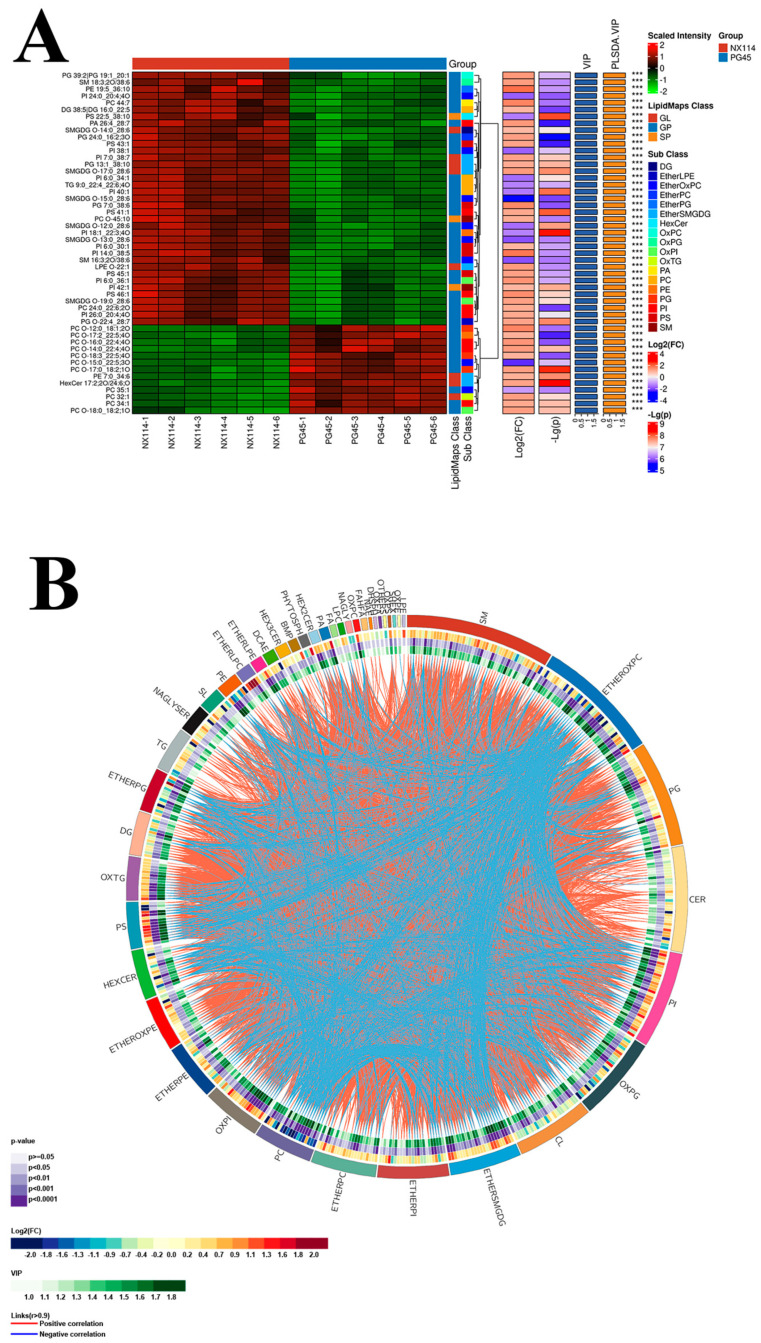
Analysis of significant differential lipids in the *M. bovis* NX114. vs. PG45 strains. (**A**) VIP Top 50 significant differential lipid hierarchical clustering results. *** VIP > 1.5. (**B**) Circos circle plot. From the outside to the inside are classification (subclasses), multiplicity of differences, *p*−value, OPLS−DA VIP, and correlation connections (positive correlation in red and negative correlation in blue).

The study aimed to understand the reasons behind differentially expressed lipids and visualize the relationship between the NX114 vs. PG45 strains and the differences in lipid expression patterns in different samples through hierarchical clustering using qualitatively significant differential lipid expression (Figure S3). We plotted the results of the VIP Top 50 hierarchical clustering of significant differential lipids (Figure 6A). The results showed that the VIP value Top 50 included 37 up-regulated differential lipid molecules (11 PI, 6 SMGDG, 5 PG, 5 PS, 3 PC, 2 SM, 1 PE, 1 DG, 1 PA, 1 TG, and 1 LPE) and 13 down-regulated differential lipid molecules (11 PC, 1 HexCer, and 1 PE). To further understand the interrelationships of lipid molecules during biological changes, the correlations between significant differential lipid molecules were presented in the form of correlation coefficient matrix heatmaps based on the Pearson correlation analysis method (Figure S4a). The results showed a positive correlation between nine significant differential lipid molecules up-regulated in the Top10 of the NX114 vs. PG45 group (PG 13:1_38:10, SMGDG O-14:0_28:6, SMGDG O17:0_28:6, LPE O-22:1, SMGDG O15:0_28:6, PI 7:0_38:7, SM 16:3:20/38:6, and PI 24:0_20:4:40) were positively correlated with each other, and the one significant differential lipid molecule that was down-regulated (PE 7:0_34:6) was negatively correlated with the other nine differential lipid molecules (Figure S4b; https://doi.org/10.6084/m9.figshare.26316751.v1). Circos chordal plots were used to visualize the existence of correlation relationships between the two lipid subclasses and lipid molecules, and the results showed that in the differential sublipid classes, sphingosine, SM, PG, and Cer were mostly up-regulated and mostly positively correlated, whereas EtherOxPC and PC were mostly negatively correlated with other lipid molecules (Figure 6B).

### 3.5. Differences in Metabolic Pathways

**Figure 7:**
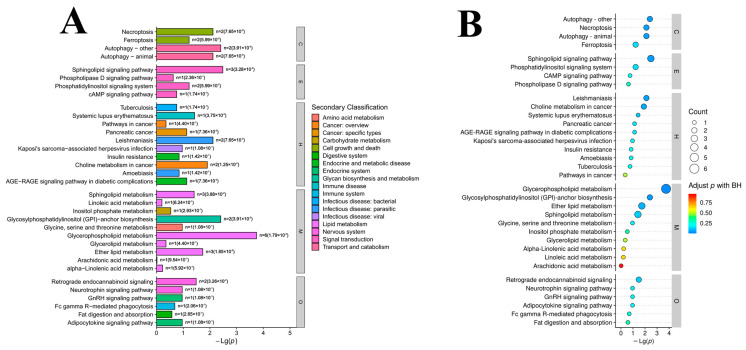
KEGG analysis of *M. bovis* NX114. vs. PG45 strains. (**A**) KEGG enrichment analysis of differential lipids. (**B**) KEGG pathway bubble diagram of differential lipids.

Lipids in organisms work together to fulfill their biological roles through a sequence of molecule coordinates. A KEGG pathway analysis can provide a systematic understanding of the function of *M. bovis* lipids and the interconnections between lipid molecules. We used the LIPEA tool to analyze the KEGG pathway enrichment of differential lipid molecules in the NX114 vs. PG45 groups, and significantly different lipids in the comparative groups were associated with 34 pathways, of which 14 differential lipid molecules had KEGG ID (Table S4) in the glycerophospholipid metabolism *(p* < 0.001), sphingolipid metabolism, and ether lipid metabolism (*p* < 0.05; Lipid metabolism); and the glycosylphosphatidylinositol (GPI)-anchor biosynthesis (*p* < 0.01; glycan biosynthesis and metabolism pathway) pathways were the most highly and significantly enriched (Figure 7A,B).

## 4. Discussion

Lipidomics is the systematic identification and analysis of lipids and the molecules that interact with them in living organisms in order to understand the structure and function of lipids and then to reveal the lipid metabolism and the physiological and pathological processes of living organisms [19]. However, no lipid-related information on *M. bovis* strains has been reported. We first looked at the lipidomes of *M. bovis* clinical strain NX114 and international standard strain PG45 at the logarithmic growth stage in this study. We achieved this by using both non-targeted and quantitative analyses. Combined with bioinformatics analysis, the relative content and species distribution of *M. bovis* lipids were characterized. To screen and identify the differential lipids, explore the potential characteristic lipids of *M. bovis*, and speculate on the changes in lipid metabolism and key pathways related to *M. bovis*.

Mycoplasmas are intermediate in size between bacteria and viruses and do not have a nucleus and organelles [20]. By electron microscopy, we can clearly observe the cell membrane structure with relatively low electron density lipids comprising the intermediate layer. In this study, *M. bovis* lipids were identified based on UPLC-Q-Exactive Orbitrap MS to consist of six major lipid classes, of which GP and SP are two important classes. The basic scaffolding of the cell membrane is composed of a phospholipid bilayer that maintains a stable cellular structure, which consists of two major classes, glycerophospholipids and sphingolipids [21,22]. Phospholipids are major components of the plasma membrane and serve not only as structural molecules but also as signaling molecules for transmitting information across the phospholipid bilayer [7,23]. In addition, fatty acids, as important components of cell membranes, play a very important role in maintaining cell structure and permeability [24]. The present study showed that GP and SP are the main components of *M. bovis* lipids and that Cer, PG, SM, TG, HexCer, EtherOxPC, and OxPG are important components of the cell membrane of *M. bovis*. The diversity of the *M. bovis* lipid structure may endow lipids with a variety of important biological functions, such as information recognition and transmission, energy conversion, and material transport. However, there are still 34 lipid molecules that have not been characterized and need to be further identified and analyzed.

This study also revealed several differential lipid molecules based on OPLS-DA model screening and analyzed the characteristic lipids between the clinical strain and the standard strain by considering the lipid species and their relative contents. Among them, only the relative contents of PC and CL were mainly significantly down-regulated. Moreover, SM and PI were found to be the two most abundant and significantly different lipids. Moreover, the clustering analysis visualized the expression pattern of lipids in both *M. bovis* strains, revealing a trend of up-regulation for SM and PI, and a down-regulation trend for PC. Based on the Pearson correlation analysis method to further understand the lipid molecule interrelationships, it was intriguing that PC was mostly negatively correlated with other lipid molecules.

SM is a lipid involved in the formation of cell membrane structure and is the most abundant sphingolipid in cells and, together with cholesterol and glycerophospholipids, constitutes the bulk of the biofilm system [25]. SM is a signal molecule that mediates important physiological processes such as cell growth, proliferation, migration, and death. Its level is crucial for cell function. At the same time, abnormal sphingolipid metabolism is related to a variety of diseases [26]. In addition, PI and its derivatives are important components that make up cell membranes and organelle membranes and are indispensable components for maintaining normal membrane fluidity and function [27]. Different PIs have different contents in different organelles and play important and different roles in different intracellular membrane systems and in material transport, lipid metabolism, and viral infection [28,29,30,31]. They are also a building block for the manufacture of inositol polyphosphates, sphingolipids, and glycophosphatidylinositols [32]. It was shown that structurally diverse and differential lipid molecules exist in different isolates of *M. bovis* and that SM, PI, CL, and PC are potentially characteristic lipids of *M. bovis*.

Then, by analyzing the metabolic pathway enrichment of *M. bovis* differential lipids, we found their metabolites were mainly enriched in seven lipid metabolic pathways, with glycerophospholipid metabolism (*p* < 0.001) and glycosylphosphatidylinositol-anchor biosynthesis (*p* < 0.01) being the most significant. Glycerophospholipids include various subclasses of lipids such as PE, PC, PI, and PS [33,34]. In this study, there were lipid molecules mapped by PE (C00416), PS (C02737), PI (C00350), CL (C05980), and PC (C00157, C00350) enriched in GP metabolism, where PE, PS, and PI showed generalized up-regulation, and CL and PC showed generalized down-regulation. It has been found that the glycerophospholipid PE is present in the outermost layer of the *Mycobacterium tuberculosis* cell membrane and that *M. tuberculosis* releases lipid molecules, such as PE and PI, into the host, contributing to the persistence of infection [35,36]. It is suggested that there are differences in lipid molecules transported extracellularly via the glycerophospholipid metabolism pathway between different isolates of *M. bovis*. It is possible that different *M. bovis* isolates have different carriers of glycerophospholipid for transport from the intracellular to the extracellular environment. In addition, alterations in the glycerophospholipid metabolism pathway may be one of the major pathways involved in the pathogenesis of pneumonia caused by *Mycoplasma pneumoniae* infection [7]. After infection of the host, the lipid bilayer of the cell membrane is susceptible to biological membrane fusion, which leads to changes in receptor recognition sites in the cell membrane, ultimately affecting the regulation of intercellular communication and metabolism [37]. Glycerophospholipids were significantly altered in the plasma of patients with *M. pneumonia*, and the enrichment of glycerophospholipid and sphingolipid metabolism pathways suggests that *M. pneumoniae* infection leads to membrane fusion damage and dysregulated lipid metabolism [7]. On the other hand, dysregulation of glycerophospholipid metabolism correlates with the severity of *M. pneumoniae* infection and the immune damage it causes [7,37]. It is suggested that the glycerophospholipid metabolism pathway may serve as a potential pathogenetic indicator and may mediate the interaction between *M. bovis* and its host through the regulation of lipid metabolism.

This study focused on the lipid metabolism of *M. bovis* strains cultured in vitro. There are certain limitations of our finding; for example, we did not analyze the lipid metabolism of the strains following host infection. It is necessary to start from the alteration of the lipid metabolism of the host regulated by *M. bovis* in the subsequent studies to further explore the relationship between lipid metabolism and the virulence and pathogenicity of *M. bovis* and to reveal the mechanism of latent infection of *M. bovis* in the host through the lipid level alterations, which will be important for the further revelation of the mechanisms of lipid-related pathogenesis and the development of new anti-pneumonia drugs and attenuated vaccines for *M. bovis.*

## 5. Conclusions

In conclusion, non-target lipids in *M. bovis* NX114 vs. PG45 were looked at in this study using a lipidological method based on UHPLC-Obitrap MS technology. First, a total of 1476 lipid species spanning 65 subclasses were detected. Second, a total of 562 differential lipid molecules, including 17 lipid subclasses with significant differences in relative content, were identified. Bioinformatics analysis was performed that examined the content and species distribution of these lipids, which explore the characteristic differentially expressed lipids of *M. bovis* with respect to the possible mechanisms underlying the differences in lipid metabolism and key pathways in these two strains.

## Data Availability

Data will be made available on request.

## Supplementary Materials

The following supporting information can be downloaded at: https://www.mdpi.com/article/10.3390/vetsci11110577/s1. Figure S1: Histogram with standard error of the percentage (%) of the combined peaks of lipid subclasses of *Mycoplasma bovis*. Figure S2: Volcano diagram of *Mycoplasma bovis* differential lipid molecules. Figure S3: Hierarchical clustering plot of significantly different lipids of *Mycoplasma bovis*. Figure S4: Heat map of correlation coefficient matrix of significantly different lipid molecules of *Mycoplasma bovis* (a: Top 300, b: Top 10) Table S1: The number of lipids identified for *Mycoplasma bovis*. Table S2: Lipid subclasses identified for *Mycoplasma bovis*. Table S3: Significantly different Lipid species of *Mycoplasma bovis* between the NX114 and PG45. Table S4: KEGG pathway analysis of differential lipids in *Mycoplasma bovis*.
