# Supplementary figures and images for: Ultra-High-Performance Liquid Chromatography–Orbitrap-MS-Based Untargeted Lipidomics Reveal Lipid Characteristics of a Clinical Strain of Mycoplasma bovis from Holstein Cow
Source: Vet Sci. 2024 Nov 18;11(11):577. doi: 10.3390/vetsci11110577 (PMC11598879; doi:10.3390/vetsci11110577)

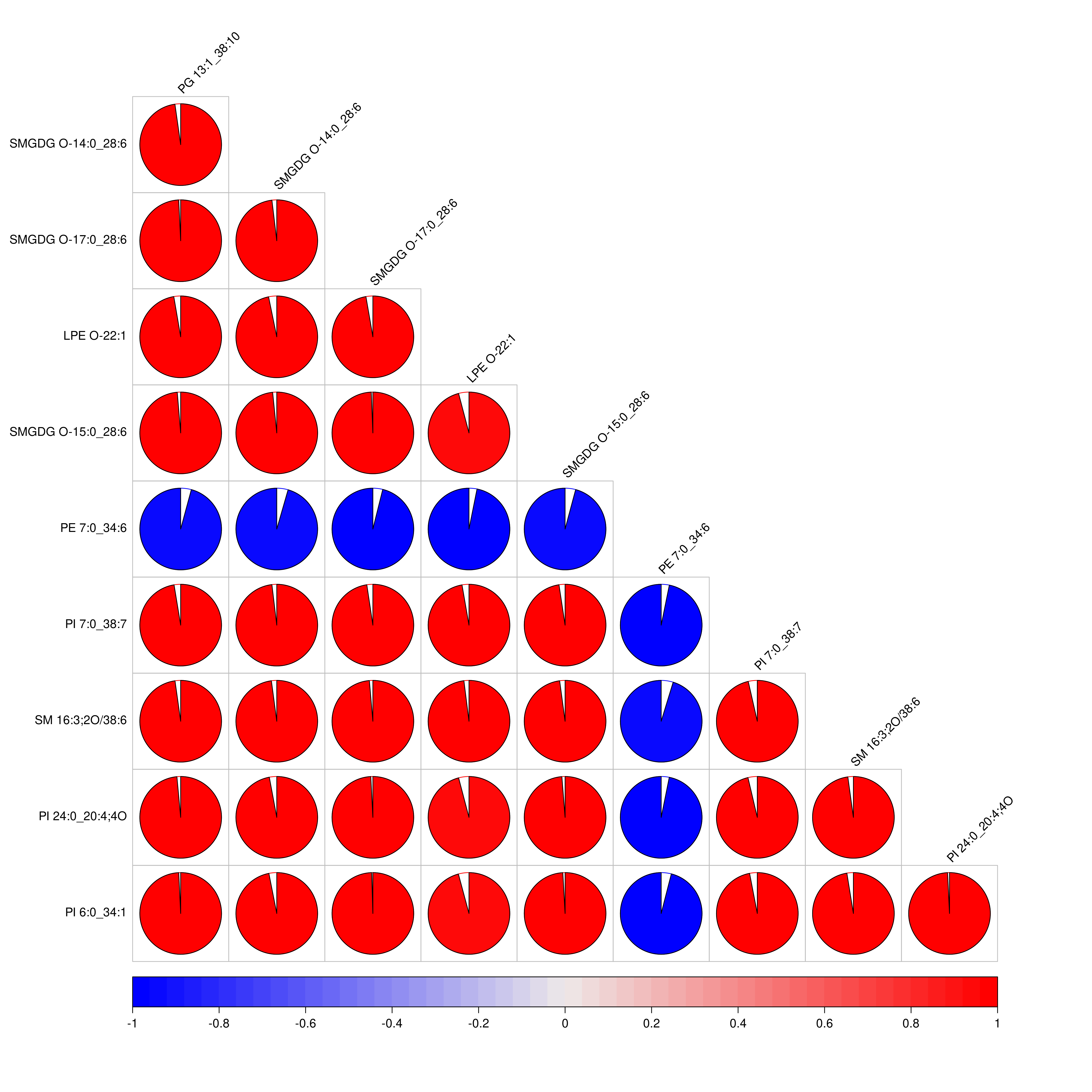

Supplement: Supplementary file 1 [file vetsci-11-00577-s001.zip › Supplementary material/Figure S4b.png]

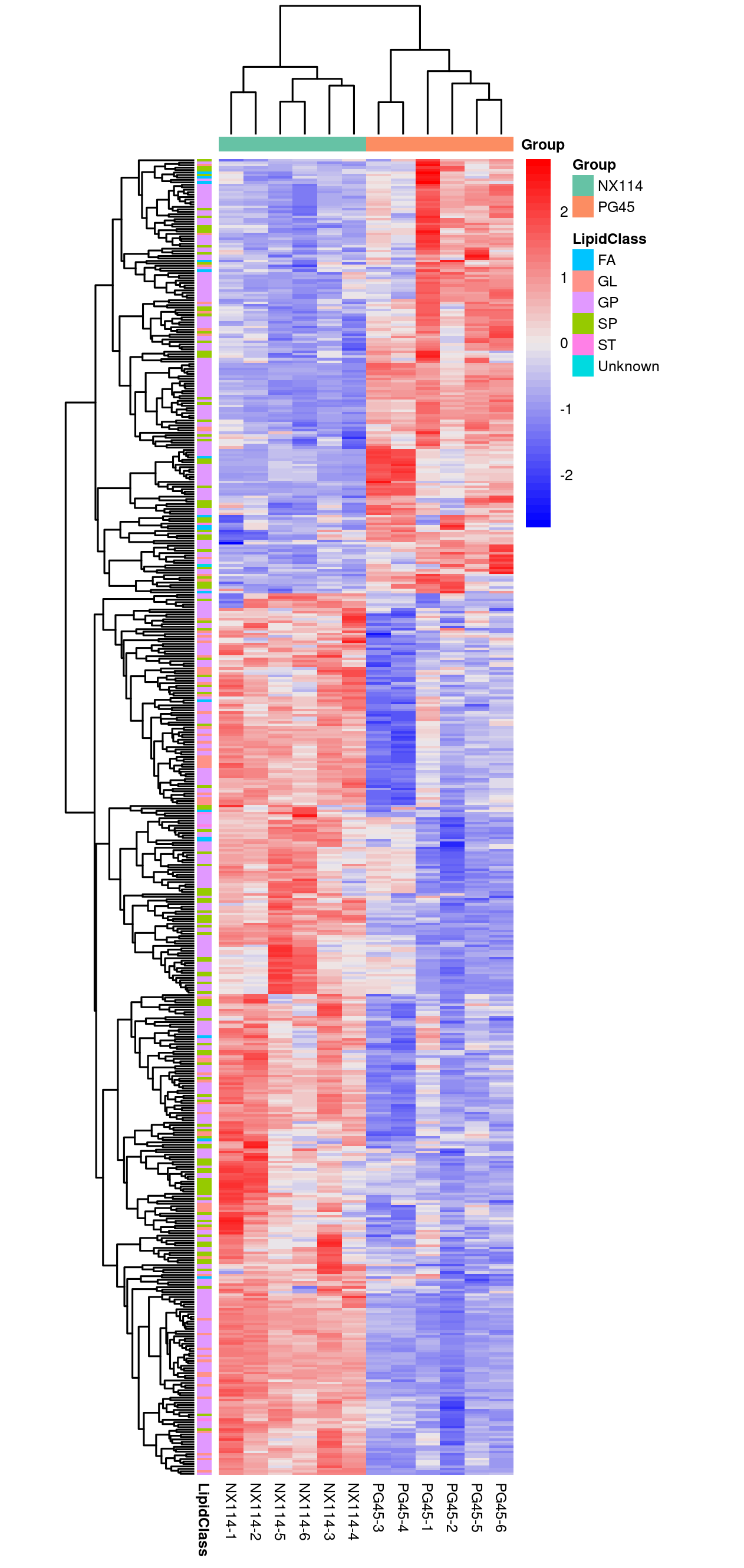

Supplement: Supplementary file 1 [file vetsci-11-00577-s001.zip › Supplementary material/Figure S3.png]

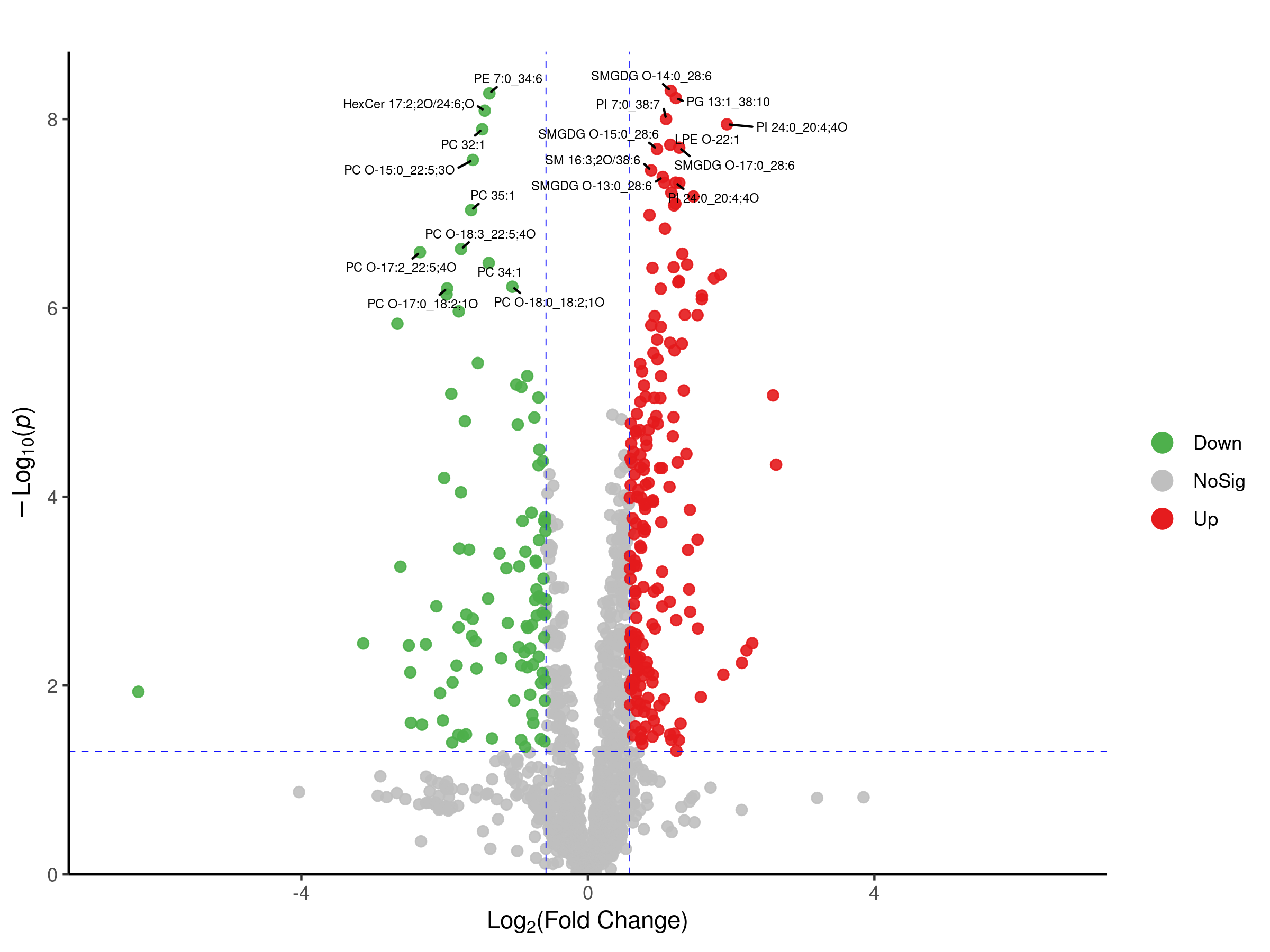

Supplement: Supplementary file 1 [file vetsci-11-00577-s001.zip › Supplementary material/Figure S2.png]

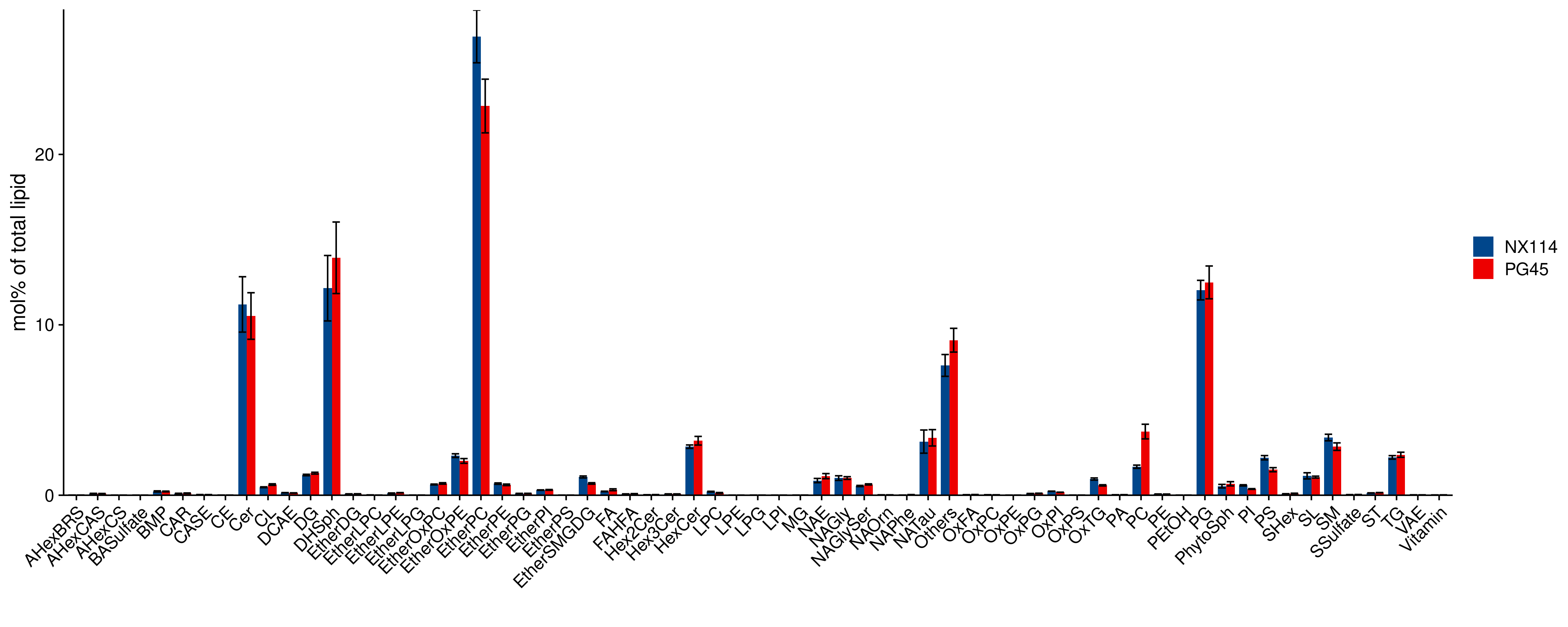

Supplement: Supplementary file 1 [file vetsci-11-00577-s001.zip › Supplementary material/Figure S1.png]
